# Post-Molding Shrinkage, Structure and Properties of Cellular Injection-Molded Polypropylene

**DOI:** 10.3390/ma15207079

**Published:** 2022-10-12

**Authors:** Artur Kościuszko, Mateusz Rojewski, Bartosz Nowinka, Filip Patalas

**Affiliations:** 1 Department of Manufacturing Techniques, Faculty of Mechanical Engineering, Bydgoszcz University of Science and Technology, Kaliskiego 7, 85-796 Bydgoszcz, Poland; 2 Gebo Technic-Engineering Ltd., Jeżynowa 21, 86-005 Zielonka, Poland

**Keywords:** polypropylene, cellular injection molding, sink marks, post-molding shrinkage, mechanical properties

## Abstract

Cellular injection molding is a common method of modifying polymer materials aimed at reducing the sink marks on moldings’ surfaces while reducing their weight. However, the dimensions of polypropylene (PP) samples as well as their mechanical properties after the injection molding process change as a result of re-crystallization. Knowledge of dimensional accuracy and awareness of the change in mechanical properties of products during conditioning are very important aspects in the polymer processing industry. The aim of this study was to assess the changes in the value of processing shrinkage and the size of the sink marks of porous PP moldings depending on the degree of porosity and the time since they were removed from the injection mold cavity. Studies of the structure and mechanical properties of moldings were carried out after several conditioning time intervals. The maximum conditioning time of samples was 840 h at 23 °C. Based on the analysis of the test results, it was found that the cellular injection molding process with the holding phase reduces the nucleation of gas pores, which results in a smaller reduction of sink marks than in the case of samples produced without the holding phase. However, PP moldings with a porosity degree equal to 8.9% were characterized by a higher shrinkage value after 1 h of conditioning, compared to moldings with porosity equal to 3.6%. The extension of the conditioning time also resulted in an increase in the value of linear shrinkage and the properties determined during tensile tests of solid and porous samples. Furthermore, in the case of samples with the highest porosity, the impact strength was reduced by about 30% after 840 h of conditioning compared to results obtained after 1 h.

## 1. Introduction

Injection molding is one of the most commonly used methods of thermoplastics processing. During the technological process, the plasticized material is introduced under pressure to the injection mold cavity. Reducing the temperature of the material causes a gradual decrease in the volume of injection moldings, which is the effect of thermal expansion and phase transformations [1,2]. In order to compensate for shrinkage losses, a holding phase is carried out.

The value of the processing shrinkage depends on many factors, including the type of polymer material, the geometry of the injection molded part, the mold cavity design, as well as the processing parameters [3,4,5]. Compared to semi-crystalline polymers, amorphous polymers are characterized by greater dimensional stability. During the cooling of semi-crystalline polymers, an ordered crystalline structure is formed, which results in a step reduction in the volume of the material in the mold [6]. The value of the primary shrinkage, i.e., the shrinkage determined after 16–24 h from removing the moldings from the cavity, can be controlled by the injection process parameters. For example, increasing the temperature of the injection mold from 20 °C to 80 °C may result in an increase in the value of the primary shrinkage of PP moldings [7]. The reduction of shrinkage can also be obtained as a result of increasing the holding pressure as well as extending the holding time, which was confirmed by Solanki on the example of gears manufactured in various process conditions [8].

Insufficient compensation for shrinkage losses results in defects in injection moldings, such as sink marks and shrinkage voids. Sink marks, i.e., depressions on the surface of injection moldings, occur mainly in the areas of accumulation of polymer material, for example on the opposite side of the reinforcement ribs [9]. The effect is a local increase in volumetric shrinkage, drawing the surface layer inside [10]. When the outer layer of the molding is resistant to shrinkage forces, holes/bubbles called shrinkage voids may form inside the core [11]. The anisotropy of processing shrinkage, i.e., differences in the value of shrinkage measured in different directions, is caused by the deformation of injection moldings [12,13,14].

The introduction of various types of fillers (talc, chalk, glass fibers) into the polymer matrix results in an increase in the dimensional accuracy of moldings [15,16,17]. In previous studies [18], Kościuszko showed that the processing shrinkage of a PP composite containing 20 wt. % of silica waste was less than 10% lower compared to the unfilled matrix. It should be also emphasized that the improvement of the dimensional stability of the filled injection moldings is also accompanied by a change in thermal and mechanical properties. PP composite containing 10 wt. % of silica can have a 20% higher tensile modulus value compared to unmodified PP [19]. However, the tensile strength and impact strength of the material deteriorated. More favorable effects in reducing shrinkage are possible when using glass fibers as a filler. Wu et al. [20] showed that the introduction of 20 wt. % glass fibers to PP matrix allowed for a reduction of the shrinkage measured 24 h after removing from the injection mold by more than 80%.

Another way to reduce processing shrinkage such as sink marks as well as voids is porous injection molding [21]. In practice, physical porous technologies are used, based on introducing gas in a supercritical state into a plasticized material [22,23]. Chemical blowing agents are also used, which, after dispersing in the polymer at the processing temperature, decompose with gas release [24,25]. Bociąga showed [26] that a greater reduction of sink marks and voids can be achieved with porous injection by increasing the amount of blowing agent used and the temperature of the plasticized material. Higher injection temperature facilitates filling the cavity of the injection mold during the holding phase, at the same time enabling an intensive porosity process which favors the elimination of the sink marks in the moldings. Besides sink marks reduction, porous injection allows for reduction the apparent density of the material, and thus the weight of the manufactured products. Llewelyn [27] showed that with the use of a chemical blowing agent in the amount of 5 wt. % it is possible to reduce the weight of PP by about 11%. In addition, porous injection allows the holding phase to be shortened or to be completely eliminated. In this case, the role of the holding pressure is taken over by the gas pressure inside the growing pores [28].

It should be emphasized that the geometry of injection moldings made of semi-crystalline materials, whose glass transition temperature is lower than the working temperature and is not stable after removing them from the injection mold and cooling to the ambient temperature. The change in dimensions of injection moldings may proceed even up to 1000 h after their removal from the injection mold, and the value of secondary shrinkage depends on the conditions of storage as well as exploitation [29]. This is confirmed by the results of Revilla-Diaz [30], who stated that the shrinkage of PP injection moldings reached a greater value after 48 h since their removal from the injection mold compared to the measurement carried out immediately after demolding. This effect is observed even in the case of PP moldings containing 10 wt. %. fillers such as calcium carbonate and glass fibers. Yue showed [31] that conditioning PP samples for 250 days resulted in an increase in stress at yield of about 20% for samples produced by compression molding. Based on previous studies [7], it was found that the shrinkage value of PP moldings may increase by 0.12 percentage points between 24 h and 504 h after removing the moldings from the injection mold, which is the result of secondary crystallization. Changes in the dimensions of moldings as a function of conditioning time are an indicator of structural changes in the material, which also result in a change in its mechanical properties. For example, Young’s modulus of the samples increased by about 20% in the same period (from 24 h to 504 h).

So far, many studies on the effect of the blowing agent amount and porous injection molding parameters on the sink marks reduction, structure and mechanical properties of PP samples were published. However, the literature lacks a description of changes in the geometry and mechanical properties of moldings as a function of conditioning time. The aim of this study was to assess the dimensional accuracy (processing shrinkage and size of sink marks) of porous moldings depending on their porosity and the time that has elapsed since their removal from the injection mold. Additionally, studies were carried out on the influence of secondary crystallization on the change of thermal and mechanical properties of the obtained samples.

## 2. Materials and Methods

### 2.1. Materials

**Figure 1:**
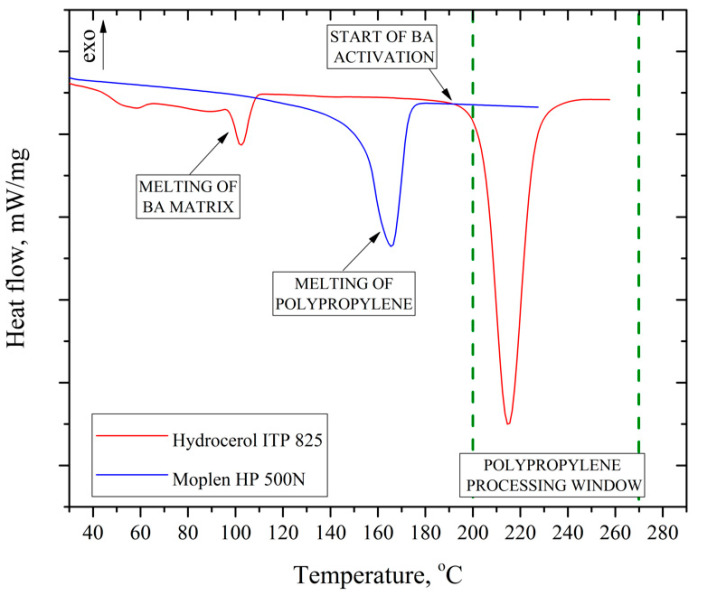
DSC curve of PP and blowing agent (CBA) recorded during heating.

An isotactic PP with the trade name Moplen HP500 N (Basell Orlen Polyolefins, Płock, Poland) was used to conduct the experiment. The melt flow rate (MFR) of the material was 12 g/10 min (230 °C/2.16 kg). The average value of Young’s modulus (*E*) and the stress at yield measured 24 h after demolding were 1020 MPa and 26.11 MPa, respectively. These values were clearly lower than those declared by the manufacturer in the datasheet. A commercial endothermic blowing agent (CBA) in the form of a concentrate under the trade name Hydrocerol ITP 825 by Clariant (Muttenz, Switzerland) was used as a modifier in the research. The active substance content in the modifier was 40% by mass. The initial temperature of the blowing agent’s activation was about 200 °C. The end of the decomposition of the active substance took place at a temperature of about 225 °C, which can be clearly seen in the curve attached in Figure 1 recorded during the differential scanning calorimetry (DSC) test. The characteristics of the selected blowing agent allowed for effective cellular injection in the temperature range of the PP “processing window”.

### 2.2. Samples Preparation

**Figure 2:**
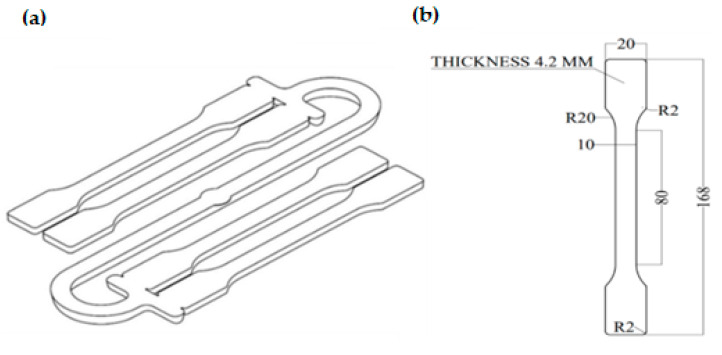
Model of the injection mold cavity (**a**) and a single test sample (**b**).

The test samples were made by injection molding using an Engel e-victory 110 (Schwertberg, Austria) machine with a mold closing force of 1100 kN, a maximum injection volume of 154 cm^3^ and a screw diameter (D) of 35 mm. The machine was equipped with a four-cavity injection mold with a cold runner, allowing the production of universal test samples (dog-bone shape) with a geometry compliant with the ISO 3167:2014 standard (type A). The model of the molding and a single sample are shown in Figure 2a,b.

The test program included the production of solid PP samples and samples with a porous structure. In order to obtain samples with various degrees of porosity, PP was modified with a blowing agent in the amount of 3 wt. %. The injection process was carried out with a variable holding time. Solid PP samples were obtained with a holding time of 21 s. Injection molding of PP with a chemical blowing agent and the holding time of 4 s resulted in obtaining samples with a degree of porosity equal to 3.6% (PP-C4). The porosity value was determined based on the measurement of the solid part density and the apparent density of porous moldings. As the effect of the injection process with the blowing agent without the holding phase, samples with a porosity equal to 8.9% (PP-C0) were obtained, which allowed for a weight reduction of about 8.2%. The recorded weight reduction was close to the value obtained by Llewelyn [26] for PP moldings (8.3%) with Trexel’s TecoCell H1 blowing agent dosing 2% by weight. The cooling time was adjusted to the holding time in such a way that the actual cooling time (sum of the holding time and the set cooling time) was equal to 45 s. Signatures, variable processing parameters and selected characteristics of the obtained samples are presented in Table 1.

**Figure 3:**
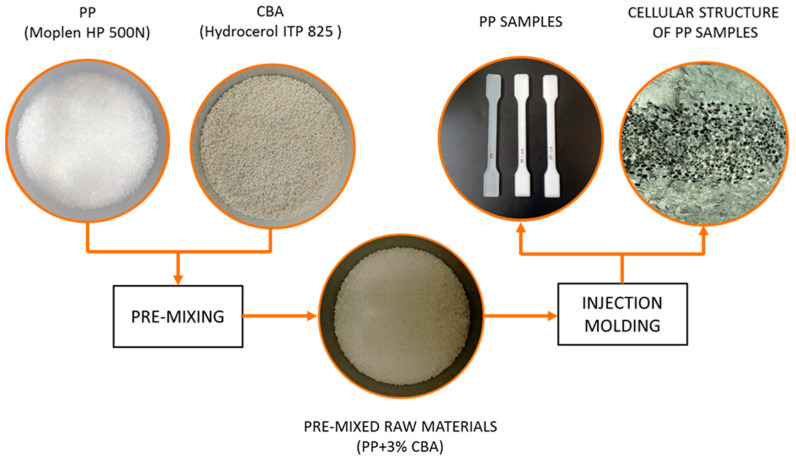
Sample preparation process.

The temperature of the injection mold was 20 °C and was controlled by the HB-160Z1 thermostat from HB-Therm AG (Sankt Gallen, Switzerland). Water was used as a cooling medium. The injection temperature was equal to 230 °C and was selected in such a way to obtain the highest possible degree of porosity of the polymeric material. Constant parameters of the sample injection process are summarized in Table 2. The obtained samples were conditioned for 840 h at 23 °C and RH = 50%. A diagram showing the sample preparation process is shown in Figure 3.

### 2.3. Measurements of Melting Behavior

Thermal properties of PP samples were determined by Differential Scanning Calorimetry (DSC) using the DSC 214 Polyma apparatus from Netzsch (Selb, Germany). The samples weighing 8–10 mg were heated to 220 °C in a nitrogen atmosphere at a rate of 10 °C/min. After two minutes of heating, the samples were cooled at the rate of 10 °C/min. The test was carried out for one sample of each test series conditioned for 1 h and 840 h. The degree of crystallinity (*Xc*) was determined with the following formula:

(1) $$X_{C}=\frac{\Delta H}{\Delta H_{C}}\times 100\%$$

where Δ*H* is the measured enthalpy of melting and Δ*H_C_* is the melting enthalpy of the 100% crystalline PP. The value of 209 J/g was adopted as the enthalpy of melting of PP with 100% degree of crystallinity [32].

### 2.4. Optical Sink Marks and Structure Analysis

Optical analysis of the sink marks size and sample structure was performed using a Keyence VHX-7000 digital microscope (Osaka, Japan). The device was equipped with a focusing lens with a possible magnification of 100–1000x. One hundred individual photos were taken between the highest and lowest points and then superimposed to obtain precise photos of the structure. The measurements of samples thicknesses were taken in the cross-section of the moldings in the middle of their length. Measurements were carried out at the temperature of 23 °C for 5 samples from each series, which were conditioned for 1 h, 168 h, 336 h and 840 h, respectively. In the case of porous samples, the size of 50 pores was measured in order to assess an average pore size in the core region of the sample.

### 2.5. Linear Shrinkage Measurements

The linear shrinkage (S) was determined on the basis of sample length measurements carried out with the Mitutoyo Quick Vision Apex CNC optical measuring center (Takatsu-ku, Kawasaki, Japan). Measurements were performed at 23 °C for samples conditioned for 1 h, 24 h, 48 h, 168 h, 336 h and 840 h, respectively. The test was performed for 10 samples from each series. The shrinkage value was calculated as the difference between the length of the moldings (*L*) and the length of the molding cavity (*L*_0_ = 168 mm) using the following equation:

(2) $$S=\frac{L_{0}-L}{L_{0}}\times 100\%\;$$

### 2.6. Measurements of Tensile Properties

The tests of mechanical properties during the static tensile test were carried out using the Z030 testing machine by Zwick/Roell (Ulm, Germany), according to the standard ISO 527-1:2020-01. The device was equipped with mechanical sample holders, a mechanical extensometer and a measuring head with a nominal force of 30 kN. The distance between holders was 116 mm and the initial spacing of the extensometer was equal to 50 mm. When determining the modulus of elasticity, the samples were tested at a speed of 1 mm/min and 50 mm/min while determining the stress and strain at the yield point. The test was carried out at a temperature equal to 23 °C for 10 samples from each series, which were conditioned for 1 h, 24 h, 48 h, 168 h, 336 h, 840 h, respectively.

### 2.7. Measurements of Dynamic Mechanical Properties

The dynamic thermomechanical analysis was carried out using the DMA GABO EPLEXOR 500N apparatus by Netzsch (Selb, Germany). Samples with dimensions of 40 × 10 × 4 mm, conditioned for 1 h and 840 h, respectively, were subjected to static and dynamic tensile loads. The test was carried out with a constant strain equal to 0.5% and 0.1%, for static and dynamic loads, respectively. The initial distance between the sample holders was 20 mm. The frequency of dynamic loads was 10 Hz. Measurements were conducted in the temperature range from 40 °C to 80 °C for one sample from each series. The heating rate was 2 °C/min.

## 3. Results and Discussion

### 3.1. Crystalline Structure

**Figure 4:**
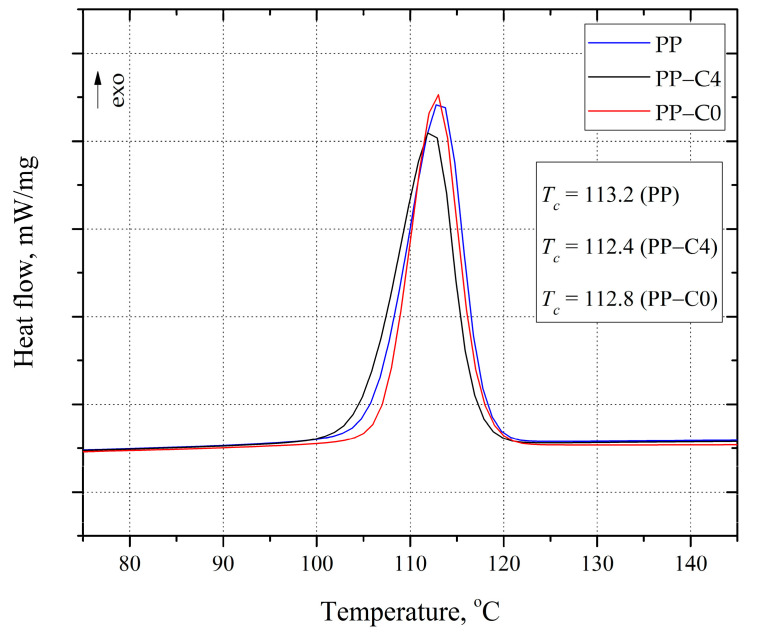
DSC crystallization curves of test samples.

Based on the analysis of the DSC curves recorded during the cooling of PP samples (Figure 4), it was found that the residues after thermal decomposition of the blowing agent did not cause heterogeneous nucleation of the modified PP. This is indicated by the lack of a crystallization temperature (Tc) shift of the PP-C4 and PP-C0 samples compared to the solid PP sample. The crystallization temperature of all tested samples ranged from 112.4 °C to 113.2 °C.

**Figure 5:**
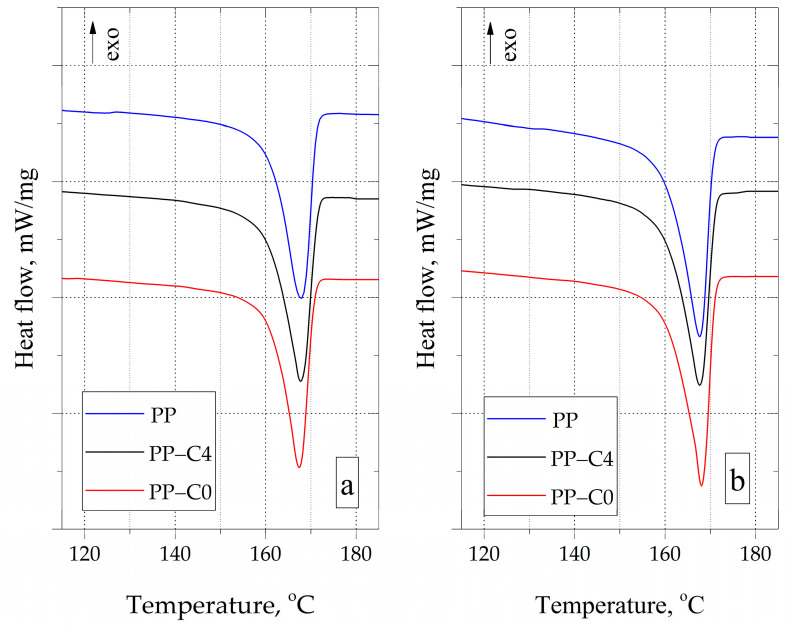
DSC melting curve of PP samples conditioned for 1 h (**a**) and 840 h (**b**).

Modification of PP with a chemical blowing agent did not cause any significant changes in the course of the curves recorded during the heating (Figure 5). The melting point of all samples that were tested after 1 h of conditioning ranged from 167 °C to 168 °C. The crystallinity degree of the solid sample and samples with a porous structure was approximately 39%, which results from similar values of the melting enthalpy. After 840 h of conditioning, an increase in Xc for all types of materials was recorded. The recorded values ranged from 41.9% for PP-C4 to 43.0% for PP-C0. The exact values of the parameters tested for each of the compositions are presented in Table 3.

### 3.2. Sink Marks and Porous Structure

**Figure 6:**
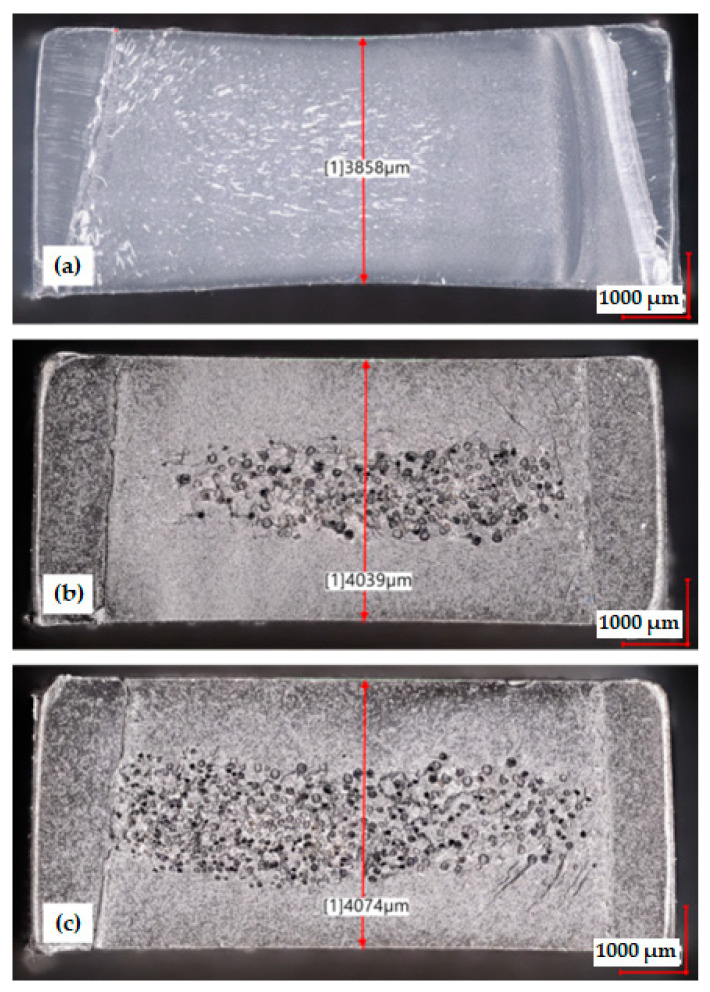
Distribution of pores in the cross-section for each of the series 168 h after them from the injection mold: (**a**) PP sample (**b**) PP-C4 sample (**c**) PP-C0 sample.

Figure 6a shows an example of a PP sample conditioned for 168 h, in which the sink marks on both sides of the surface are clearly visible. This effect results from the lack of compensation of shrinkage losses during the injection process, which may be caused, among other things, by a high thickness of the molded part or too low a temperature of the injection mold, which results in a quick solidification of the gate. The average thickness of the PP samples, measured in the cross-section of the moldings in the middle of their length after 1 h from their removal from the injection mold cavity, was equal to 3875 µm (Table 3). The thickness gradually decreased over time. After 168 h and 840 h of conditioning, this dimension reached an average value of 3856 µm and 3852 µm, respectively. The increase in the depth of the sink marks is the effect of secondary shrinkage caused by the crystallization of PP outside the injection mold cavity [7].

The expected effect of chemical cellular injection of PP was the reduction of the size of the sink marks on the surface. Figure 6c clearly shows that the use of the blowing agent allowed for the elimination of sink marks despite the lack of the holding phase during sample production. A reduction of the sample mass by about 9% was also achieved. It was possible thanks to the gas pressure inside the growing pores, which was described by Palutkiewicz [28]. The average thickness of the PP-C0 samples after 1 h of conditioning was 4085 µm and was 2.7% smaller compared to the dimensions of the injection mold cavity. A clear change in the size of the PP-C0 samples was observed only after the first conditioning phase (168 h). With the passage of time, the moldings did not change their thickness significantly.

Surprisingly, the samples that were produced using a holding time of 4 s (PP-C4) had a lower thickness compared to the PP-C0 samples. For example, the average thickness of the PP-C4 samples after 1 h of conditioning was 4045 µm, i.e., 3.7% less compared to the dimensions of the injection mold cavity. The observed phenomenon is a consequence of the holding pressure, which, on the one hand, allowed us to introduce a larger amount of plasticized material into the mold cavity, but limited the possibility of nucleation of pores in the structure of the molded part. This is confirmed by the photographs of the structure of samples PP-C4 and PP-C0, presented in Figure 5c and Figure 6b, respectively. The average pore size in the core area was equal to 97 ± 20 μm in the case of PP-C0 and 93 ± 19 μm for PP-C4 samples. These values do not differ significantly and the changes in pore size over time were not noticed. Despite similar pore size, the area of porous structure in the core of PP-C0 samples is clearly larger compared to the area occupied by the pores in PP-C4 samples, which determines the difference in porosity of the obtained materials. Therefore, it can be concluded that during the injection process of PP with a chemical blowing agent, in order to obtain the greatest possible reduction of sink marks, it is advisable to shorten the holding phase as much as possible. Moreover, samples with lower porosity (PP-C4) showed a greater change in thickness during conditioning. The difference between the thickness of the PP-C4 samples after 1 h and after 840 h of conditioning was about 0.6% and was the same as for unmodified PP. In the case of PP-C0 samples, the difference was only 0.3%. This effect is most likely due to the presence of a greater amount of solid PP in the cross-section of PP-C4 samples, which tended to change in volume during conditioning. The exact thickness results of the samples for each series are shown in Table 4.

### 3.3. Linear Shrinkage

**Figure 7:**
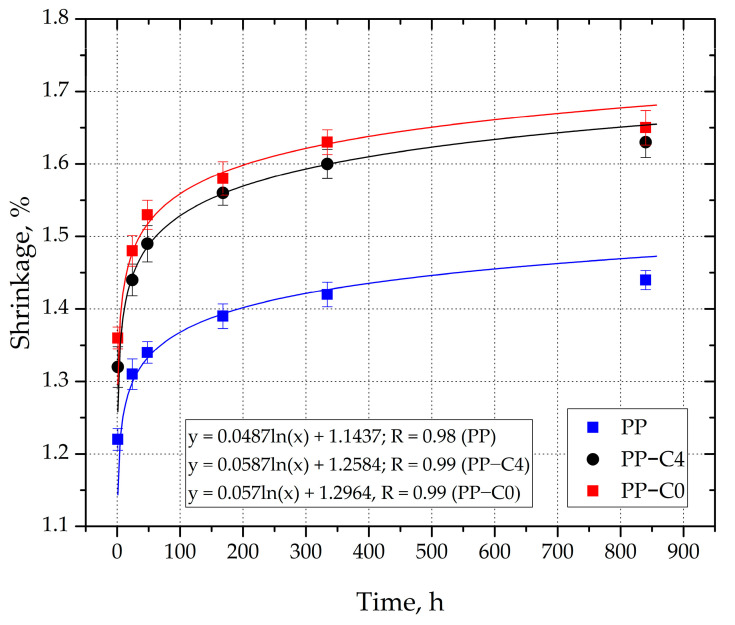
Changes of processing shrinkage values as a function of the conditioning time.

Despite the fact that chemical porosity reduced the sink marks, the value of linear shrinkage of PP-C4 and PP-C0 samples was unexpectedly higher compared to solid PP, regardless of the conditioning time after which the measurements were taken (Figure 7). Moreover, 24 h after removing the moldings from the injection mold, the linear shrinkage of PP samples (primary shrinkage) reached the value of 1.31%, while in the case of PP-C4 and PP-C0 samples the values were higher by 9.9% (S = 1.44%) and 13% (S = 1.48%), respectively. The length of the samples gradually decreased during the conditioning. For example, the shrinkage value of the PP sample after 840 h was equal to 1.44%, which corresponds to a reduction in length compared to the measurement after 24 h by 0.22 mm. Such large changes in the dimensions of moldings during conditioning may limit the use of this type of materials in construction areas.

Moreover, after 840 h of conditioning, the difference between the shrinkage of porous and solid samples increased to the values of 13.2% (PP-C4, S = 1.63%) and 14.5% (PP-C0, S = 1.65%), respectively. It can therefore be concluded that PP moldings with a porous structure are also characterized by a slightly higher value of secondary shrinkage measured along the flow path of the material in the injection mold. Moreover, there is no linear relationship between the weight of the molded parts and the shrinkage value. Despite the application of a 4 s holding phase, the course of changes in the value of linear shrinkage of PP-C4 samples as a function of conditioning time was similar to the course determined for PP-C0 samples, where the holding phase was not applied. However, in this case, the application of 4 s of pressure resulted in a slight reduction in the value of shrinkage measured along the flow path.

The increase in the value of the processing shrinkage for all types of samples along with the extension of the conditioning time is the effect of the increase in the degree of crystallinity, as it was previously described. Moreover, it can be concluded that the gas pressure inside the expanding pores allows for the reduction of sink marks; however, it is not sufficient to compensate shrinkage losses along the flow path of the material in the mold. The effect of porous injection is to increase the accuracy of mapping the surface of the moldings in relation to the shape of the injection mold cavity. However, striving to significantly reduce the weight of the moldings as a result of shortening the time of the holding phase may result in a reduction in the dimensional accuracy of the moldings measured along the flow path. Therefore, at the stage of designing the mold cavities dedicated to the injection process of PP with a chemical blowing agent, a higher value of linear shrinkage should be assumed than for solid PP. When designing moldings that are supposed to have a porous structure, attention should also be paid to the specific type of shrinkage anisotropy. In this case the difference between the shrinkage measured along the flow path of the material and the transverse shrinkage are determined for the thickness of the moldings. After 840 h of conditioning, the PP-C0 shrinkage reached the value of 1.65%, while the difference between the thickness of the part with reduced sink marks and the dimensions of the injection mold cavity was 2.7%.

### 3.4. Mechanical Properties

**Figure 8:**
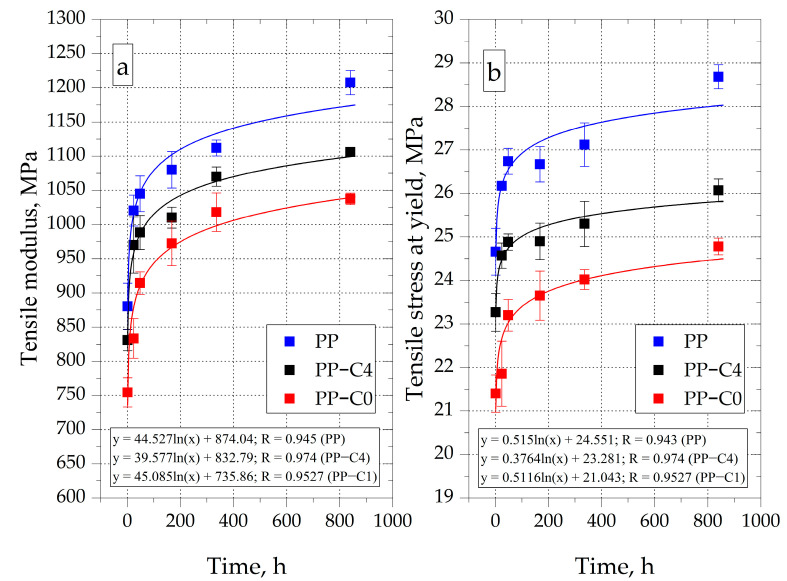
Changes of tensile modulus (**a**) and tensile stress at yield (**b**) as a function of the conditioning time.

The Young’s modulus of moldings made of unmodified PP, 1 h after their removal from the injection mold cavity, was equal to 880 MPa (Figure 8a). After 24 h of conditioning, an increase in the E value to 1020 MPa was registered, and after 840 h to 1207 MPa. During the secondary shrinkage, i.e., from 24 h after the end of the injection cycle until the end of the experiment (840 h), an 18% increase in the PP elasticity modulus was recorded. Similar favorable changes in the value of E during the conditioning were also recorded in previously conducted studies [7].

PP samples with a porous structure were characterized by a lower value of the Young’s modulus compared to the solid material. The E value of PP-C0 samples after 1 h of conditioning was equal to 754 MPa, which is about 14% less compared to the value determined for PP after the same conditioning time. Along with the extension of the conditioning time, as in the case of solid PP, a gradual increase in the value of the elastic modulus of PP-C0 samples was recorded. After 840 h, the Young’s modulus of this material was equal to 1037 MPa, which is also 14% less than in the case of unmodified PP.

The stress at yield values (σ_y_) for all types of samples also increased with conditioning time (Figure 8b). For example, σ_y_ of PP-C0 samples after 1 h from demolding was 21.4 MPa, while after 840 h of conditioning it was 24.8 MPa. This represents an increase of approximately 15.8%, which is similar to the solid PP (16.3%). However, the stress at yield of the porous specimens was lower compared to that of the solid material. Similar effects were observed in Palutkiewicz’s research, where the tensile strength of PP samples deteriorated by about 22% as a result of dosing 2 wt. % of chemical blowing agent [33]. In the case of samples with the highest porosity (PP-C0), σ_y_ was about 14% lower than for PP, regardless of the conditioning time. 

**Figure 9:**
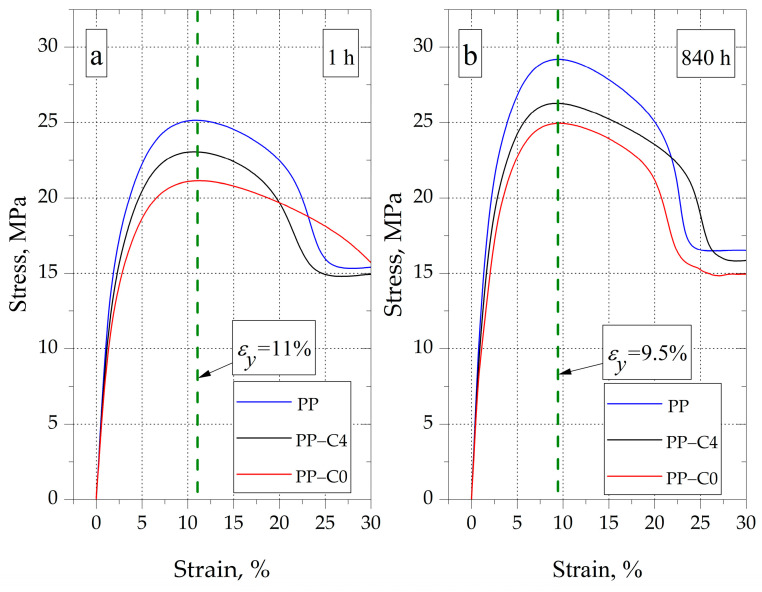
Examples of tensile curves of PP samples; after 1 h of conditioning (**a**), after 840 h of conditioning (**b**).

The effect of changes in the modulus of elasticity and stress at yield due to the increase in the degree of crystallinity of PP is also clearly visible in the example tensile curves recorded after 1 and 840 h of conditioning presented in Figure 9a,b. Moreover, the indicator of the strengthening effect is also the change of the deformation value at the yield point (ε_y_). The average registered value of this parameter for samples conditioned for 1 h, regardless of the degree of porosity, was about 11%. After 840 h of conditioning, the ε_y_ decreased to about 9.5%.

The lower value of E and σ_y_ of porous PP samples results from the reduction of the amount of material in the sample volume. Improvement of the mechanical properties of samples during conditioning is the result of an increase in the value of the degree of crystallinity in the material. It is also worth noting that the course of changes in the value of the modulus of elasticity and the stress at yield as a function of the conditioning time of the PP-C4 samples is between the curves determined for the PP and PP-C0 samples. Therefore, it can be concluded that the values of E and Rm, unlike the processing shrinkage, significantly depend on the mass of the moldings, and thus on their porosity.

### 3.5. Impact Strength

During the impact tests, it was observed that the PP samples did not break until 336 h after their removal from the injection mold cavity. The impact strength during this time reached the value of 150 kJ/m^2^ (Table 5). Extending the conditioning time to 840 h reduced the impact strength by 15% (128 kJ/m^2^). The increase in the brittleness of the material is the result of changes in the crystalline structure of PP. The reduction of the impact strength as a function of the conditioning time was also recorded in the case of samples with a porous structure; however, the PP-C4 and PP-C0 samples were broken after each of the analyzed time intervals. The impact strength of porous samples after 1 h of conditioning was equal to 82 kJ/m^2^ for PP-C4 and 67 kJ/m^2^ for PP-C0. After 840 h from removing the samples from the injection mold, the impact strength reached the values of 62 kJ/m^2^ and 47 kJ/m^2^, respectively, representing a reduction of 24% (PP-C4) and 29% (PP-C0). It is also worth emphasizing that the impact strength of porous samples, regardless of the parameters of the injection process, was significantly lower compared to solid PP. After 840 h of conditioning, these differences were 52% for PP-C4 and 64% for PP-C0, respectively. On this basis, it was found that the impact strength of PP with a porous structure does not significantly depend on the value of the porosity. The very presence of pores, i.e., voids in the material, causes crack propagation, lowering the impact strength.

**Figure 10:**
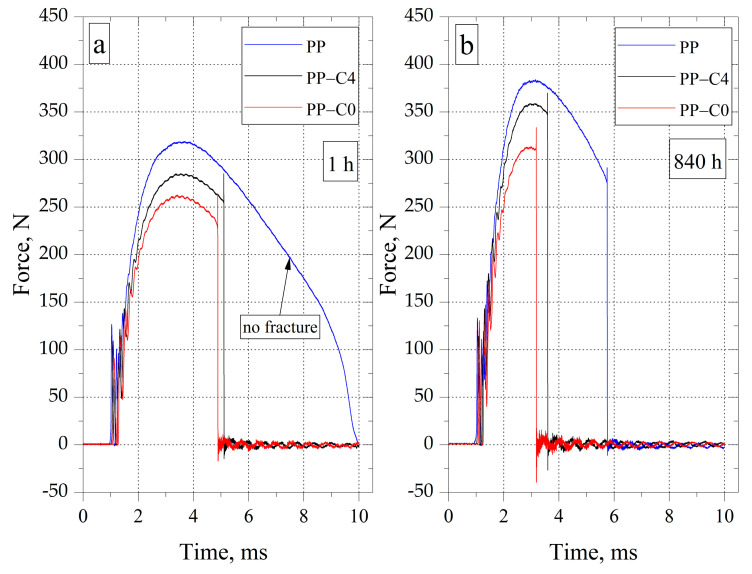
Dependence of the force on the time of fracture for the tested samples during the conditioning time equal to: (**a**) 1 h (**b**) 840 h.

Another dependence was observed in the case of changes in the maximum force recorded during the impact test as a function of the conditioning time. For each type of sample, the maximum force escalated with increasing conditioning time. The increase in maximum force results from the increase in stress at the yield point of the tested materials as a function of time. The lack of combination of the increase in the maximum force on the impact strength value results from the increase in the content of the crystalline phase characterized by greater stiffness in PP, which results in the cracking of the samples after a shorter time of force application (Figure 10a,b). In the case of the PP-C0 sample conditioned for 1 h, the cracking occurred after less than 5 ms of the test. Conditioning for 840 h shortened the time of destruction to about 3 ms. Awareness of the increase in brittleness of PP with a porous structure along with the conditioning time to remove the molded parts from the injection mold is crucial for the right material for a given application. Based on the analysis of the test results, it can be concluded that passing the quality control tests by PP moldings, which are carried out immediately after the production process or within the next several hours, does not have to guarantee that the product will carry the assumed loads already at the operating stage.

### 3.6. Viscoelastic Properties

**Figure 11:**
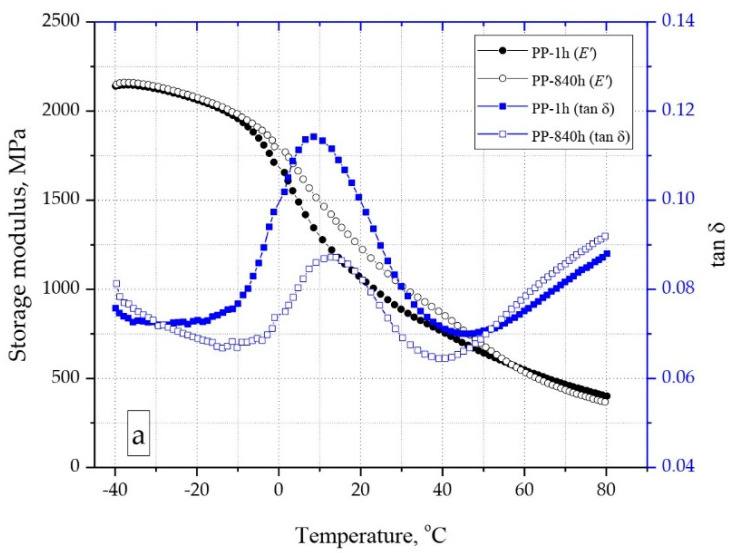

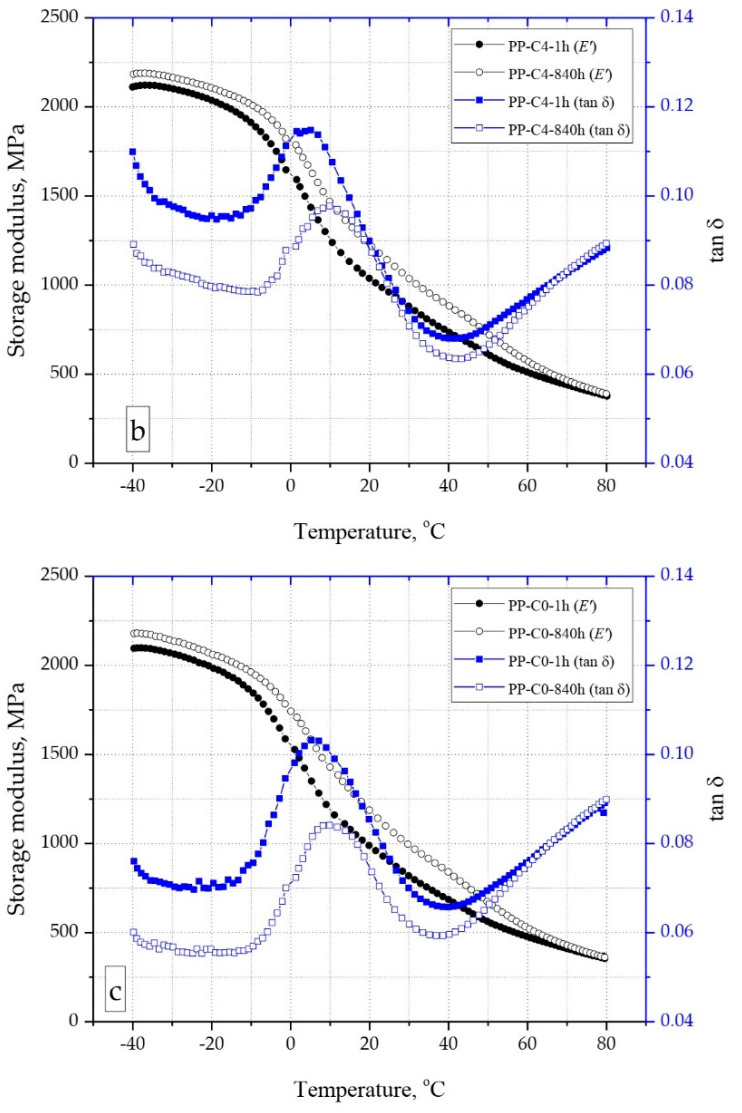
Storage modulus and mechanical loss factor of polypropylene samples: PP (**a**), PP-C4 (**b**), PPC0 (**c**).

Based on the results of research on viscoelastic properties (Figure 11a–c), it was found that PP moldings with a porous structure are characterized by a lower value of the storage modulus (E’) compared to solid PP samples. The differences were recorded over the entire temperature range of the test. As in the case of Young’s modulus, the increase in porosity of the moldings resulted in a decrease in the E’ value. For example, at the temperature of 23 °C after 1 h of conditioning, the storage modulus of PP samples (1006 MPa) was higher by about 8% compared to the PP-C0 samples (930 MPa). After 840 h of conditioning, a significant increase in the value of the conservative modulus was recorded, in particular in the temperature range from −10 °C to 50 °C, i.e., in the range close to the operating temperature. The increase in E’ between 1 h and 840 h of conditioning for the temperature of 23 °C was from 16% for PP to 20% for the PP-C0 samples.

The increase in the degree of crystallinity of PP, observed along with the extension of the conditioning time of the samples, also caused an increase in the glass transition temperature (T_g_), which was determined as the maximum on the curve of dissipation factor (tan δ). For PP, a T_g_ shift from 8.5 °C to 12.5 °C was recorded. Samples with porous structure, regardless of the degree of porosity, after 1 h of conditioning, were characterized by a lower glass transition temperature compared to PP. For example, for PP-C0 samples, a change in glass transition temperature from 5 °C to 10 °C was noted. Therefore, it can be concluded that the operating temperature range of PP moldings, both solid and porous structures, becomes narrower with the passage of time. The effect of the increase in the glass transition temperature may be an increase in the brittleness of the material, already observed at a slightly higher temperature than in the case of direct measurements or after several hours after removing the moldings from the injection mold.

## 4. Conclusions

The results of the conducted tests confirmed that the cellular injection allows for the reduction of sink marks on PP moldings. Better accuracy of the injection mold geometry was obtained in the case of samples produced without the holding phase. The effect of the holding pressure caused the introduction of a larger amount of plasticized polymer into the injection mold cavity. As a result, the nucleation of pores was limited and thus the porosity of the moldings was reduced while maintaining the average pore size at a constant value. The effect of the increase in porosity is a proportional reduction of the modulus of elasticity and the stress at the yield of the obtained materials. The presence of gas bubbles in the PP structure also reduces the impact strength of the material. However, in this case, it was found that even a relatively low degree of porosity causes a significant reduction in this parameter.

Based on the research, it was found that despite the significant reduction of sink marks, chemical porosity does not compensate for shrinkage losses measured along the flow path of the material in the injection mold, resulting from the reduction of time or elimination of the holding phase. The value of shrinkage measured along the flow path of the material in the mold is not linearly correlated with the degree of porosity; however, the application of the holding phase results in a reduction of its value. Along with the lengthening of the conditioning time, an increase in the shrinkage value of both solid and porous moldings was recorded, while it was found that the moldings with a porous structure were characterized by a slightly higher value of secondary shrinkage. The change in dimensions of the moldings as a function of the conditioning time is caused by an increase in the degree of crystallinity, which is also accompanied by a change in the mechanical properties of the moldings, an increase in the value of the elastic modulus, an increase in stress at the yield and a decrease in impact strength.

Knowledge about the dimensional accuracy of the processed materials is important at the stage of designing products as well as injection molds, especially in the case of constructional moldings that are combined with other elements, where high dimensional tolerances of the order of the hundredth part of a millimeter are required. On the other hand, the awareness of changes in the mechanical properties of PP moldings during conditioning is essential for the proper selection of material for a given application.

## Figures and Tables

**Table 1:** Sample markings and selected process parameters.

Signature	Holding Time [s]	Cooling Time [s]	Content of Blowing Agent [wt. %]	Samples Mass [g]	Samples Porosity [%]
PP	21	24	0	8.51 ± 0.01	-
PP-C4	4	41	3	8.19 ± 0.01	3.6
PP-C0	0	45	3	7.81 ± 0.03	8.9

**Table 2:** Constant parameters of the injection process of PP samples.

Processing Parameters	Value
Feed zone temperature, °C	190
Transition zone temperature, °C	210
Metering zone temperature, °C Nozzle temperature, °C	230 230
Mold temperature, °C Counter-pressure, MPa	20 1.5
Injection velocity, cm^3^/s	75
Holding pressure, MPa	22

**Table 3:** Thermal properties of PP samples.

Conditioning Time, h	Sample	Melting Temperature, °C	Melting Enthalpy, J/g	Xc, %
1	PP	168.0	81.59	39.0
	PP-C4	167.7	82.68	39.6
	PP-C0	167.4	81.81	39.1
	PP	167.8	88.68	42.4
840	PP-C4	167.6	87.51	41.9
	PP-C0	168.0	89.84	43.0

**Table 4:** Thickness of conditioned PP samples.

Conditioning Time [h]	Samples Thickness [μm]		
	PP	PP-C4	PP-C0
1	3875 ± 5	4045 ± 4	4085 ± 5
168	3856 ± 6	4040 ± 3	4076 ± 7
336	3859 ± 4	4036 ± 7	4074 ± 8
840	3852 ± 6	4021 ± 5	4074 ± 5

**Table 5:** Parameters recorded during impact strength measurements.

Material	Parameter	Time, h					
		1	24	48	168	336	840
PP	Impact strength, kJ/m^2^	-	-	-	-	150 ± 11	128 ± 8.0
	Max. force, N	330 ± 13	331 ± 8.0	342 ± 3.0	357 ± 10	360 ± 11	380 ± 7.0
PP-C4	Impact strength, kJ/m^2^	81.9 ± 8.3	72.2 ± 4.4	67.1 ± 5.2	63.3 ± 2.3	62.3 ± 3.6	61.8 ± 1.9
	Max. force, N	296 ± 11	319 ± 10	335 ± 8.0	338 ± 14	348 ± 8.0	370 ± 5.0
PP-C0	Impact strength, kJ/m^2^	66.8 ± 3.8	62.7 ± 6.7	61.7 ± 5.6	56.2 ± 1.8	52.8 ± 0.9	47.5 ± 1.1
	Max. force, N	249 ± 7.0	266 ± 7.0	301 ± 11	312 ± 6.0	318 ± 7.0	335 ± 3.0

## Data Availability

The data presented in this study are available on request from the corresponding author.
